# Unraveling the Molecular Complexity of Adenoid Cystic Carcinoma (ACC): A Comprehensive Exploration of Hub Genes, Protein-Protein Interaction (PPI) Networks, microRNA (miRNA) Involvement, and Drug-Gene Interactions (DGIs)

**DOI:** 10.7759/cureus.54730

**Published:** 2024-02-22

**Authors:** Roja L Karri, Manasa Bojji, Amrutha Rudraraju, Abdul Sadik Mohammad, Vamseedhar Kosuru, Sandeep Kalisipudi

**Affiliations:** 1 Oral and Maxillofacial Pathology, GSL Dental College and Hospital, Rajahmundry, IND; 2 Oral and Maxillofacial Pathology, Malla Reddy Dental College for Women, Hyderabad, IND; 3 Dentistry, Koppal Institute of Medical Sciences, Koppal, IND; 4 Pediatric and Preventive Dentistry, GSL Dental College and Hospital, Rajahmundry, IND; 5 Pediatric and Preventive Dentistry, Narayana Dental College and Hospital, Nellore, IND; 6 Pediatric and Preventive Dentistry, Lenora Institute of Dental Sciences, Rajahmundry, IND

**Keywords:** drugs, mi-rnas, hub genes, differentially expressed genes, adenoid cystic carcinoma

## Abstract

Background

Adenoid cystic carcinoma (ACC) poses clinical challenges with its unique histology and potential for perineural invasion, recurrence, and distant metastases. Recent genomic advancements have unveiled key genetic alterations in ACC, offering insights into its pathogenesis.

Aim

This study aims to unravel the intricate molecular landscape of ACC through a comprehensive analysis of gene expression patterns. By integrating data from multiple microarray datasets, the study explores differentially expressed genes (DEGs), their functional enrichment, protein-protein interactions (PPI), hub genes, microRNA (miRNA) involvement, transcription factors, and potential drug-gene interactions.

Methods

Three microarray datasets (GSE88804, GSE153002, and GSE36820) related to ACC were selected from the Gene Expression Omnibus (GEO) repository. DEGs were identified using GEO2R and further analyzed for commonalities and differences. Functional enrichment analysis, including Gene Set Enrichment Analysis (GSEA), provided insights into biological processes, cellular components, molecular functions, and Kyoto Encyclopedia of Genes and Genomes (KEGG) pathways associated with ACC. PPI networks and hub genes were identified using the Search Tool for the Retrieval of Interacting Genes/Proteins (STRING) (STRING Consortium, Lausanne, Switzerland) database and Cytoscape (Cytoscape Consortium, California, United States). The study also explored miRNAs, transcription factors, and potential drug-gene interactions.

Results

The integrated analysis revealed 339 common upregulated and 643 downregulated DEGs in ACC. Functional and pathway enrichment analyses unveiled the involvement of these genes in critical cellular processes, signaling cascades, and pathways. The PPI network, comprising 904 nodes and 4139 edges, highlighted the complexity of interactions. Hub genes, including KIF11, BUB1, and DLGAP5, were identified, shedding light on their pivotal roles in cell cycle regulation. The study also identified miRNAs (e.g., hsa-mir-7-5p and hsa-mir-138-5p) and transcription factors (e.g., E2F1 and TP53) associated with ACC. Drug-gene interactions have identified potential therapeutic options, including amsacrine and rucaparib.

Conclusions

The ACC gene expression highlights a nuanced molecular landscape, identifying pivotal hub genes such as KIF11 and CDK1 as potential therapeutic targets for ACC, given their roles in cell cycle progression. The dysregulation of microRNAs and transcription factors adds complexity to ACC's molecular profile. Exploration of drug-gene interactions reveals promising therapeutic strategies, involving FDA-approved drugs such as amsacrine and rucaparib, providing avenues for personalized interventions.

## Introduction

Adenoid cystic carcinoma (ACC) is an uncommon and enigmatic malignancy with distinct clinical challenges. Originating primarily in secretory glands, notably the salivary glands, ACC accounts for 1% of malignant tumours and 10% of salivary gland neoplasms [1]. It is distinguished by its distinct histological features, which include solid, tubular, and cribriform development patterns. Despite its slow growth and characteristic histology, ACC poses a formidable threat due to its tendency for perineural invasion, recurrence, and distant metastases, which frequently involve the liver, lungs, and bones.

Recent advancements in genomic research have shed light on key genetic alterations driving ACC pathogenesis, such as the MYB-NFIB translocation observed in a significant majority of cases. These genetic insights, coupled with the identification of alterations in pathways such as Wnt/β-catenin and aberrant expression of growth factor receptors, provide a foundation for unraveling the molecular landscape of ACC [2].

Gaining insight into the complex genetic foundations of ACC improves our understanding of the disease's aetiology and creates opportunities for focused treatment interventions. The investigation of these molecular aspects may bring about a new era of precision medicine for ACC patients, providing hope for a better prognosis, treatment options, and diagnosis in the face of this difficult and enigmatic cancer. Hence, the present study aims to delve deeper into the intricate molecular landscape of ACC. Through a comprehensive analysis of upregulated and downregulated differentially expressed genes (DEGs), protein-protein interaction (PPI) networks, hub genes, microRNA (miRNA) involvement, and interactions between drugs and genes, this study aspires to unravel the complex molecular machinery that underlies the development and progression of ACC. This multifaceted approach seeks to provide a holistic understanding of the genetic and molecular intricacies governing ACC, paving the way for more precise and effective clinical interventions.

## Materials and methods

Data abstraction

From the Gene Expression Omnibus (GEO) repository, three microarray datasets (GSE88804, GSE153002, and GSE36820 related to ACC) were meticulously chosen [3]. GSE88804 comprised nine ACC tissues and seven normal tissues, while GSE153002 included 30 ACC and seven normal samples. Both datasets were based on the GPL6244 platforms (Affymetrix Human Gene 1.0 ST Array). Additionally, the data of GSE36820, which consisted of eight cancer tissues and three normal tissues, was based on the GPL571 platforms (Affymetrix Human Genome U133 Plus 2.0 Array).

DEGs identification

GEO2R was utilised in conjunction with the R packages GEOquery and limma to identify DEGs that met stringent criteria, such as p-value ≤ 0.05 and logFC > 0.5. Genes with p < 0.05 and log FC > 1 were considered upregulated DEGs, while genes with p < 0.05 and log FC < −1 were considered downregulated DEGs. Following this, the standalone user-friendly interface tool FunRich (FunRich, Germany) was utilized to analyze and interpret heterogeneous datasets, thereby facilitating the identification of common upregulated and downregulated DEGs among the three datasets, with the Venn diagram generated by FunRich aiding in visualizing overlapping gene sets [4].

Gene set enrichment analysis (GSEA)

The upregulated and downregulated DEGs underwent functional enrichment analysis using Enrichr (Ma'ayan Laboratory, United States) revealing their participation in crucial biological processes, cellular components, and molecular functions [5]. Additionally, KEGG pathway analysis provided insights into the specific pathways these DEGs are associated with, further enhancing the understanding of their role in ACC pathogenesis.

PPI network and hub genes

Subsequent analyses were based on the DEGs from the attained datasets, a PPI network was constructed using the STRING database, and hub genes were identified through Cytoscape 3.10.1 analysis employing the CytoHubba plugin [6-8].

miRNAs and transcription factors identification

The miRNet tool was utilized to identify potential miRNAs associated with the hub genes, contributing to the understanding of post-transcriptional regulatory networks in ACC [9].

Hub gene-drug interaction

The identified hub genes underwent a comprehensive investigation as potential drug targets through an in-depth analysis utilizing the Drug-Gene Interaction Database (DGIdb) (Washington University School of Medicine, United States). This extensive exploration involved examining the intricate interactions between these hub genes and existing drugs [10].

## Results

DEG’s identification

**Figure 1:**
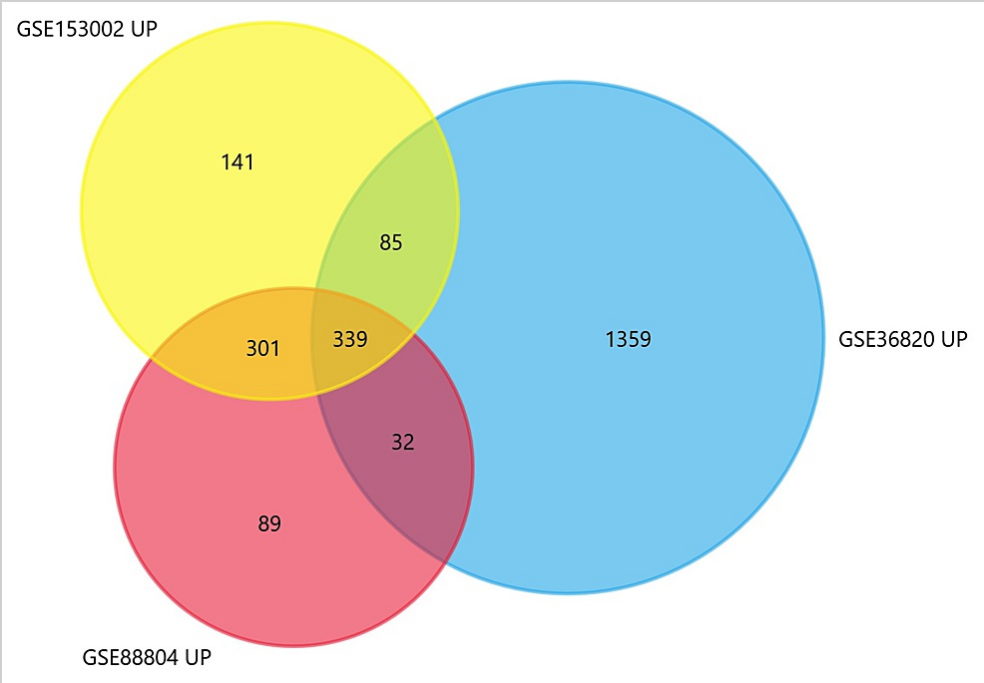
Common upregulated DEGs among datasets DEGs: Differentially expressed genes

**Figure 2:**
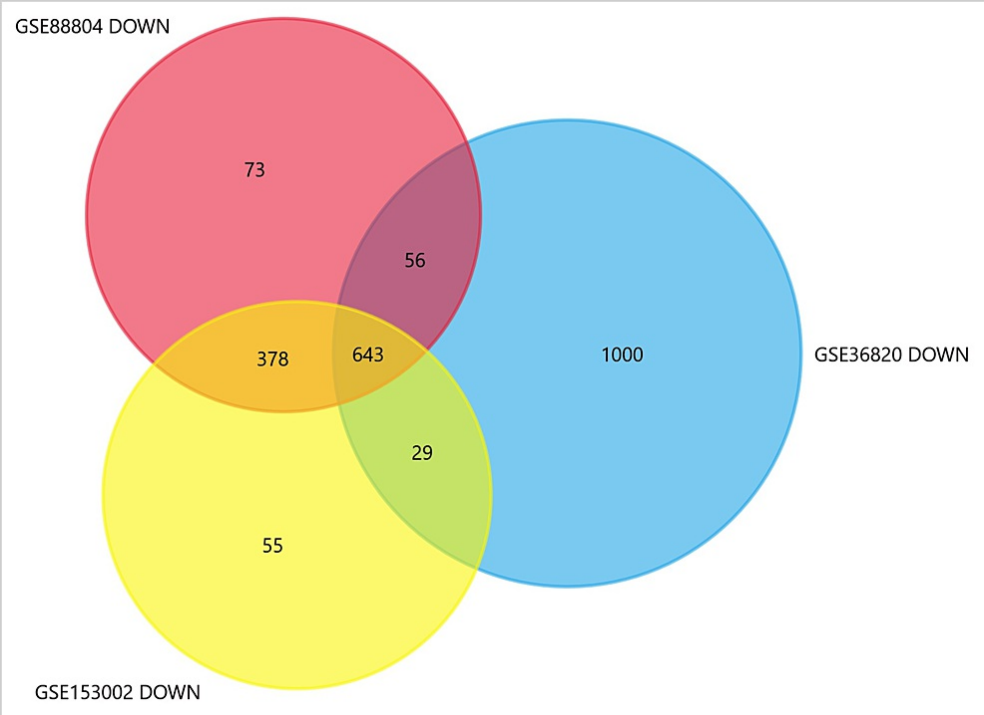
Common downregulated DEGs among datasets DEGs: Differentially expressed genes

In the integrated analysis of gene expression datasets GSE88804, GSE153002, and GSE36820, a comprehensive exploration of ACC revealed a set of 339 common upregulated and 643 downregulated genes (Figure 1 and Figure 2).

The list of upregulated and downregulated genes is given in Table 1.

**Table 1:** List of upregulated and downregulated DEGs DEGs: Differentially expressed genes

Gene Expression	Gene Symbol
Upregulated Genes	BMPR1B, STMN1, VTCN1, GABRP, SOX4, ABI2, ZNF286B/ZNF286A, EZH2, FAT1, NLN, RPGRIP1L, STK26, VCAN, TESMIN, ZWILCH, TUSC3, CDK6, HELLS, PGM2L1, FABP7, FZD7, CENPJ, KIF23, EFHD1, WEE1, ZNF682, ZNF711, EN1, PRAME, ELAVL2, POGLUT1, PAWR, KIF3A, MCM7, GINS1, BRIP1, FNDC1, ZFP1, ANLN, NUP93, TRIM37, MEX3C, ZC3HAV1L, WDHD1, WDR12, KIF22, ZNF84, C4orf46, ZNF135, IP6K2, ZNF431, AGPAT5, TBC1D32, POLE2, TP53, NUDT11, PUS7, DNAH14, MTHFD2, H2AFY2, FBXL2, PITPNM2, RAD54B, FAM83B, ANKRD50, ZRANB3, BUB1B, KDM5B, NUSAP1, LINC00665, STX6, ZFP69B, ABCA13, FANCI, BCL2, DTL, CENPK, CENPU, TOP2A, LDLRAD4, FLVCR1, RASGRP1, RPP40, BICD1, EPT1, SPDL1, UBE2T, IGSF3, TTLL4, PRLR, KIF11, KNTC1, COL27A1, JAG1, SPAST, PLK4, ATAT1, MSANTD3-TMEFF1/TMEFF1, PLXDC2, AADAT, CCNB2, TYMS, ZNF300, WNK3, GGH, CDCA7, TRIP13, AFAP1, HMGB3, SHC4, KPNA2, CKS2, ITGA9, GLMN, CENPF, FIGNL1, KIAA1524, CDC42EP3, CTTNBP2NL, SEPT4, TM4SF1, ART3, C21orf91, KNL1, MFGE8, CKAP2, PLXNB1, DNMT1, PCNA, TPX2, ZNF512, TRIM45, RAPGEF4, TMEM97, BUB1, GUCY1A3, CEP85, MCM3, PRC1, RAP2A, TRAM1L1, IQCJ-SCHIP1/SCHIP1, MTBP, KALRN, PMS2P1, MCM4, SMC4, ST3GAL4, NCAPG2, NCAPG, MND1, BRCA2, DGKH, PLEKHG4B, ARNT2, CHEK1, CCT6A, SEMA6D, SPAG5, PLCG1, MKI67, MELK, HENMT1, GCNT2, CHML, DAPK1, PTTG1, NETO2, EPHB3, TRO, TTYH1, PARPBP, ZNF260, CCDC34, PHF14, KIAA0101, SLC35F3, SKP2, DPY19L2P2, SRPK2, NHSL1, BCL2L11, ZNF519, DPY19L2, FAM198A, SPSB1, PARD6G, CEP55, ARHGEF9, CCNB1, KIF20A, YPEL1, LRRCC1, SKA3, C18orf54, CDK19, CENPI, ZNF559-ZNF177/ZNF177, CCNA2, DIAPH3, PGAP1, OBP2B, CDK1, PSD3, ZNF681, SGO2, WDFY2, NOTCH1, TIAM1, ZNF492, ZNF667, ZNF850, RFC3, ZNF670, FANCD2, ZFP82, DLGAP5, LAPTM4B, SH3PXD2B, OLFM2, MAP4K4, GPX8, TICRR, SQLE, MCM6, DCAF4, TRIM59, CENPH, SERPINE2, RAD51AP1, MOK, TBC1D1, CABYR, LAMB1, CACNB3, BAMBI, KIF14, GINS2, TTK, NUF2, DDIAS, PDE9A, KIF15, TRPS1, HEY2, RACGAP1, MID1, C5orf34, NOTCH3, DDIT4, SGO1, FZD3, ASPM, IL17RB, MICAL3, KIF18A, ZNF610, CENPE, GPSM2, ZNF738, MRC2, E2F7, OSBPL3, CERS6, GEN1, POLQ, EPHX4, GJC1, CDKN3, SHCBP1, CDC45, CDC6, TFAP2A, ELOVL6, PCSK6, ZNF124, EFNA4, APBB2, MFAP2, PBK, NEIL3, GPX7, EXO1, SERPINH1, KLHL13, MCM10, SNHG3/SNORA73A, MFAP3L, ZNF257, CYTH2, NEK2, PCNX1, MMP16, SDK2, COLEC12, CDC7, ZNF664-FAM101A/FAM101A, HORMAD1, FSCN1, CDC25B, MLC1, SPC25, LEF1, GLIPR2, COL9A1, IGFBP2, FAM69C, SCRG1, SLC9B2, HAPLN1, FAM178B, PCDHB2, ZFHX4, DSC3, NCS1, COL4A2, HAPLN3, FGFR1, LAMC2, SYCP2L, DKK1, PCDHB3, SLC2A1, NCKAP5, LOX, CDH2, NRCAM, LGR6, SPARC, KRT6B, SCG5, TACR1, FRZB, TM4SF18, DSG3, NTRK3, NID1, LOXL2.
Downregulated Genes	TESC, ADRA1A, MYRIP, BHLHA15, NXPE4, ETNPPL, FBP1, SLC31A2, HM13, AGFG2, FAM20A, CSN1S2AP, CPD, LPO, MCFD2, TM7SF2, DNASE2B, ATP2B2, SLC37A2, TBC1D30, FXYD6-FXYD2/FXYD2, ACADL, SLC9A1, AGMAT, PRUNE2, MAOB, LRRTM1, ALDH1L1, MLPH, DHRS7, PDE8B, SLC26A9, KIAA2022, ADIRF, PKDCC, RNF150, KLK1, GALNT6, FAM3D, SIDT1, SLC1A2, DNER, WIF1, SEC11C, PRH1-PRR4/PRR4, TRPV6, PAX9, MGLL, C1orf115, C1orf168, KCNJ16, ALAD, GRIA4, PDZK1IP1, FMO6P, GIPC2, DEPTOR, SLC20A2, FUT6, SEMA3G, SLC43A1, STXBP6, RAP1GAP, FGF10, CLCNKB, WWC1, SNTB1, TPD52L1, C1QTNF3-AMACR/AMACR, C8orf4, XBP1, PIK3AP1, LRG1, PDZD8, CSN2, ADRB1, PGR, FAM129A, MELTF, C4orf32, MYOC, SLC22A3, SPCS3, ZCCHC2, SH3BGRL2, ADIPOQ, BPIFA2, PIGR, TNFRSF11A, WNT5A, FAM171B, ELL2, SMCO4, KCNK6, FAM174B, KIAA0513, GNMT, GPD1, DUSP6, COL6A6, ARL4D, TPK1, SLC31A1, FGF12, PEBP4, CRACR2A, TXNDC11, ALDH18A1, NOSTRIN, SERP1, SLC16A14, SULT1C2, INPP5J, SLC39A8, DHDDS, CD55, GNE, RRBP1, TRIM8, PPP1R36, NUCB2, MEIS2, ERN1, CST5, ATP8B1, HSD11B2, GALE, CD40, EPHA2, RILPL2, PIP, FAM20C, DLK1, FUT8, PRKACB, MLKL, DMBT1, TBC1D24, SLCO4A1-AS1, GNA14, FAM3B, KIAA1024, TMED3, ZG16B, MRAP2, ACVR1C, RNF128, CST2, MAOA, PDE3B, LRP1B, CTBS, POF1B, CASP7, SEC62, PRB3, ACVRL1, KAT2B, SMAD9, FKBP11, TMEM45B, GGT6, SOAT1, PRB4, C2orf88, SIGIRR, ADIPOR1, DAPL1, EIF4EBP1, DNAJC3, ERGIC1, FAM84B, PDCD4, ETV1, ACPP, CADPS2, RRAGD, G0S2, DENND3, TSPAN13, HDAC9, PRKCH, VSIG10L, SH2D4A, BCAR3, DEFB1, SMR3A, ME3, IGSF10, MBNL3, CPEB3, CXXC5, CPT1A, ANGPTL1, DYX1C1-CCPG1/CCPG1, FNDC5, ANO4, NUPR1, DPP4, PLPP5, AQP3, BLM, CALM1, ARFGEF3, MSMB, SPR, SPATS2L, PTGER3, PGPEP1, ARHGAP26, PLIN1, SIX1, GPR160, PDE11A, PDHA1, TCEA3, RAB3D, ATG4A, TMEM62, LDLRAP1, FZD5, RAB17, ODAM, RNLS, AFF1, ACACB, ARHGAP24, GAREM1, SEC23B, KCNJ15, CFD, RGCC, TFAP2B, FAM46A, CYSTM1, SERPINB1, C9orf152, PON3, NRK, CEP85L, BACE2, CA6, ICAM3, KIAA0040, CTPS1, RASD1, ARFGAP3, SLC35F2, PLLP, MAN1A1, ACOT11, AQP5, NANS, SERPINI1, TMEM61, RSPO3, CSN3, PLA2R1, SBSPON, DHRS2, CPLX3, FUT2, RORC, PALMD, SYBU, ARHGAP5-AS1, ADH1B, ARHGAP29, CPEB4, GALNT16, GABARAPL1, GATM, FOXP2, RIN2, EIF4E3, TMC5, SLC25A20, PDE3A, ENPP3, CBX7, AOC3, SIK2, SRGAP1, DNAJB9, SPDEF, TMEM132B, SLITRK5, MPP1, ZNF827, REPS2, NUDT4P1/NUDT4P2/NUDT4, SLPI, PLTP, ADH1C, COX14, DNAJC1, NR3C2, KRT80, ALDH1A1, SLC17A5, CASP4, GATA3, SSR4, STXBP1, PARM1, CRYBG3, TRAK2, PCYOX1, ADHFE1, AACS, ACAT1, MLXIPL, CCL28, CACNB2, LINC01554, TNFSF15, TLR3, TCN1, GPD1L, HERPUD1, SPX, RBM47, MUC7, CST3, MAST4, SSR3, RUNX1T1, NIPAL2, CD14, ST3GAL6, NR3C1, MOCS2, CLIC6, LBH, FAM46C, ZFHX3, IQGAP2, ERBB4, TNFRSF19, LYZ, LMOD3, LIFR, ABLIM1, VAMP8, MAPT, TMEM181, ADGRG2, PLEKHH1, MAPK13, SQRDL, ANGPT1, ASB5, GREM2, ATP6V0A4, HSDL2, TSPAN8, MANSC1, HTN3, XDH, ESRRG, SP100, SLC13A5, SCGB3A1, LPAR3, RGN, PLCB4, ENTPD3, ADGRB3, FIGN, FOXQ1, KDELR3, RAB27B, FABP4, ACSL1, ASPA, HTN1, FRMD4B, LY9, DPYD, INSIG1, MUCL1, PHLDA1, NRXN1, PLIN2, RHOBTB3, SV2B, WDR72, DOPEY2, LAMA4, TAB2, PITPNC1, SH3BP4, COBLL1, GALNT15, DUSP1, WFDC2, MGST2, PKP2, SDCBP, HS3ST3B1, EPB41L4A, HLA-DMA, AGTR1, HOMER2, TNS1, CHCHD7, ERO1B, KIAA1324, TTLL7, KIF21A, IL6ST, NOS1, RASEF, SLC39A11, LCN2, SLCO2A1, NCEH1, CTSC, SOD2, PCK1, OAS2, LIMCH1, PLPP3, CEBPD, TAC1, CRACR2B, MUC15, TMEM99, PTGS2, ZNF704, ARL4A, RASSF5, ALDH2, TMPRSS11E, CYP4B1, PLAG1, ALDH3A2, GLRX, SYT7, PHLDB2, DPT, STATH, GAL3ST4, HHATL, IGKC, HBA2/HBA1, ZFP36, INPP5D, NDRG2, SH3BP5, PDK4, CRISP2, LRRC2, March3, MECOM, C16orf89, NAMPT, ZDHHC2, CD36, CCDC69, CDH19, CST1, HLF, TNFSF10, KLK11, GCH1, CCDC110, EXPH5, FAXDC2, ATP6V1C2, SLC40A1, RHOU, MME, CRISP3, FAM107B, CREB3L1, SYTL3, ANKRD29, LDB2, TMEM140, NCALD, NR4A2, PLCB1, SH3RF2, SP140L, FMO5, CAMK2N1, PRKAA2, NMRK1, SCNN1A, IL1B, EGF, MTUS1, ATP2A3, ATP1B1, NFIA, NEDD9, IER3, GALNT13, GGTA1P, NFKBIZ, FDCSP, GLRB, MEIS1, NEBL, GPCPD1, SPRY1, JCHAIN, STK39, SLC13A2, CTSL, CKMT2, TSPAN12, IGF1, ANO5, NQO1, PART1, CSRNP1, COL15A1, C4orf19, ZDHHC15, ABCA9, TNXB/TNXA, NRXN3, GULP1, CYP39A1, CARD6, UNC5C, PIP5K1B, HSPB8, TNFRSF17, ERO1A, SOBP, FOLR1, SLC16A7, JUNB, GCNT3, TGFBR2, TSPAN1, RGS2, NBEA, TSPAN5, PODN, PLSCR4, PDE4B, TMC4, IRF1, FBLN5, HBB, LTF, PLEKHS1, FHL1, STK17B, AOX1, SELE, TSC22D3, ELF5, CHRDL1, CXCL12, SLC15A2, AMPD1, FABP3, BSPRY, FOSB, ABCA8, IGK/IGKC, EDNRB, TSHZ2, OPRPN, ANXA1, FOLH1B/FOLH1, CXCL2, PRKG1, FOS, SCN7A, PLA2G16, STEAP4, ABI3BP, CLDN10, TCIRG1, APBB1IP, ADAMTSL3, STX19, LPAR1, KLF4, NR4A3, ITPR1, SPTSSB, CST4, CEACAM1, CA2, APOD, MCOLN3, PPARGC1A, METTL7A, ABCA6, PPP1R3C, KCNMA1, BIRC3, IL6, CFTR, VWF, SLAMF7, C5orf46, HCP5, SLC12A2, ST6GAL1, VWA5A, CYTIP, ATF3, AGR2, EMCN, LINC01140, CCL5, CPED1, CEACAM6, CYSLTR1, C3, FYB, EVI2B, FMO2, CXCL8, KLRC4-KLRK1/KLRK1, CLDN1, GBP2, FGL2, SNORD3D/SNORD3C/SNORD3B-2/SNORD3A/SNORD3B-1, LY96, EFEMP1, CD69, TNFAIP6, TXLNGY, CD52, ANK2, C7, DOCK8, HLA-DQA1, TFPI, CD53, MS4A1, PTPRC, RPS4Y1.

**Figure 3:**
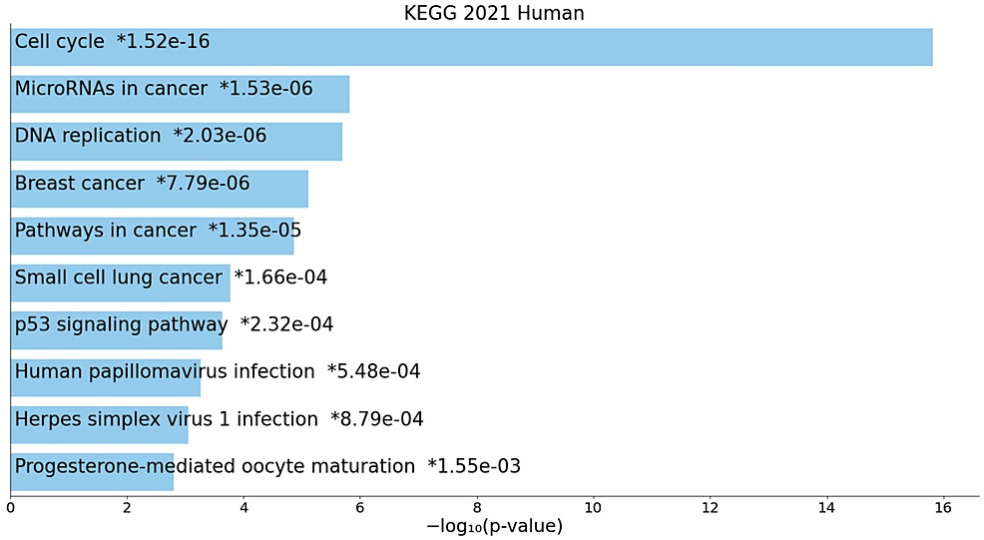
KEGG pathway for upregulated genes * p < 0.05 - statistically significant

Functional and Pathway Enrichment of DEGs:

The molecular, biological, and cellular processes associated with ACC reveal a complex landscape of dysregulated pathways. KEGG pathway analysis of upregulated genes highlights the involvement of critical signaling cascades such as the cell cycle, miRNAs in cancer, DNA replication, and various cancer-related pathways (Figure 3).

**Figure 4:**
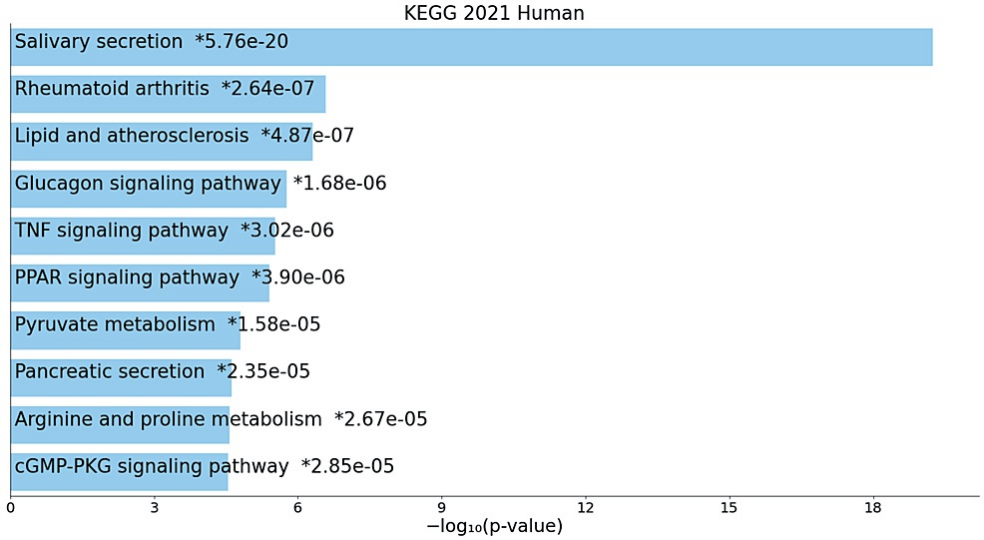
KEGG pathway for downregulated genes * p < 0.05 - statistically significant

On the other hand, KEGG pathway analysis of downregulated genes reveals diminished activities in pathways such as salivary secretion, rheumatoid arthritis, lipid and atherosclerosis, glucagon signaling, tumour necrosis factor (TNF) signaling, peroxisome proliferator-activated receptor (PPAR) signaling, pyruvate metabolism, pancreatic secretion, and arginine and proline metabolism, signifying a global influence on various cellular processes (Figure 4).

**Figure 5:**
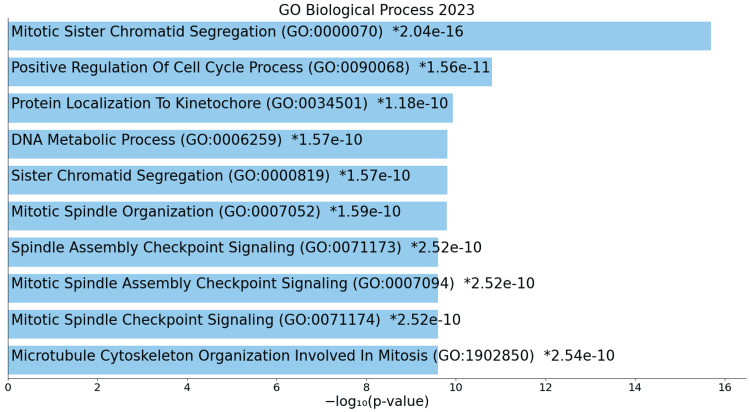
GO biological process for upregulated genes * p < 0.05 - statistically significant GO: Gene ontology

At the biological process level, upregulated genes show aberrant regulation in key cellular activities, including mitotic sister chromatid segregation, positive regulation of cell cycle processes, and spindle assembly checkpoint signalling (Figure 5).

**Figure 6:**
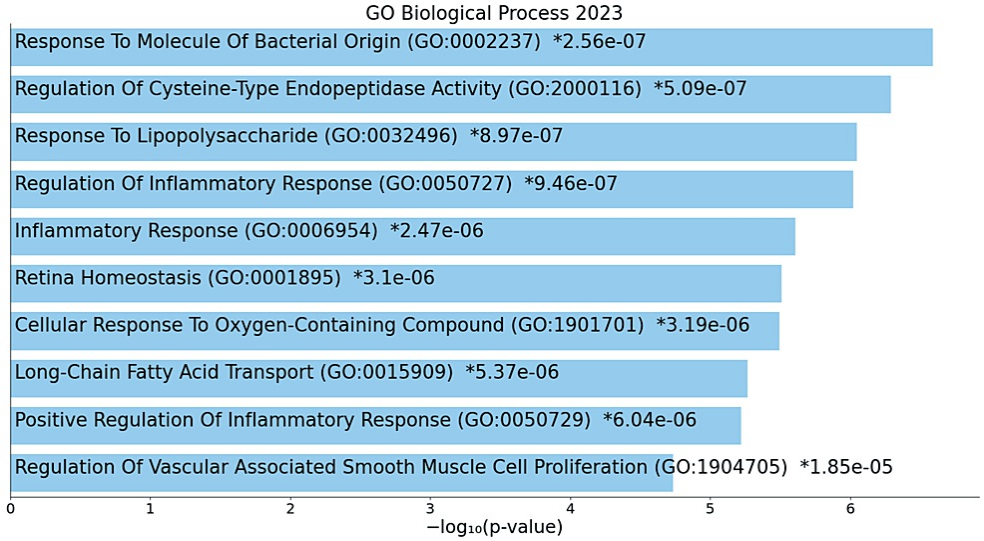
GO biological process for downregulated genes * p < 0.05 - statistically significant GO: Gene ontology

Conversely, downregulated genes exhibit reduced responses to external stimuli and impaired regulation of inflammatory responses, highlighting a diminished capacity for adaptive cellular behaviours (Figure 6).

**Figure 7:**
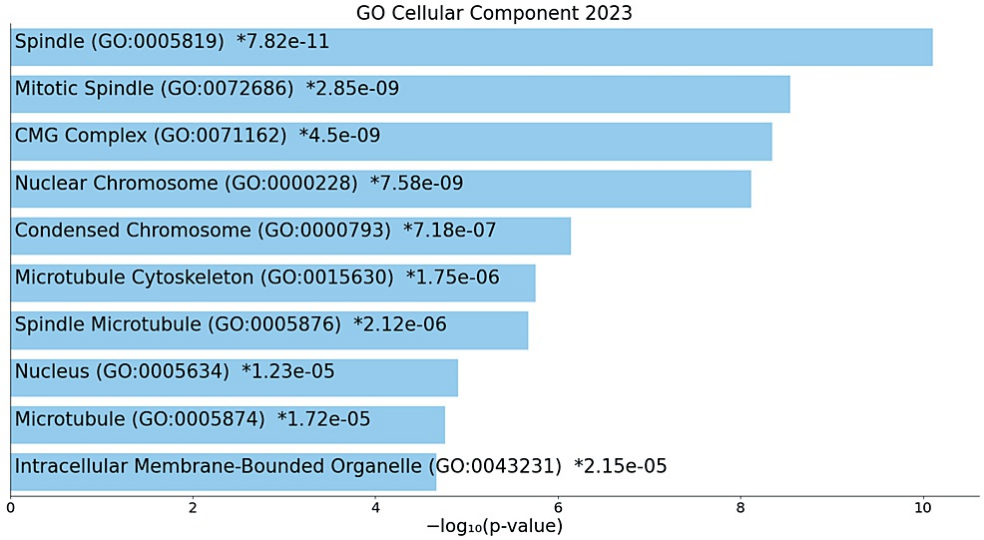
GO cellular process for upregulated genes * p < 0.05 - statistically significant GO: Gene ontology

On the cellular front, upregulated terms encompass structures such as the spindle, mitotic spindle, and nuclear chromosome (Figure 7).

**Figure 8:**
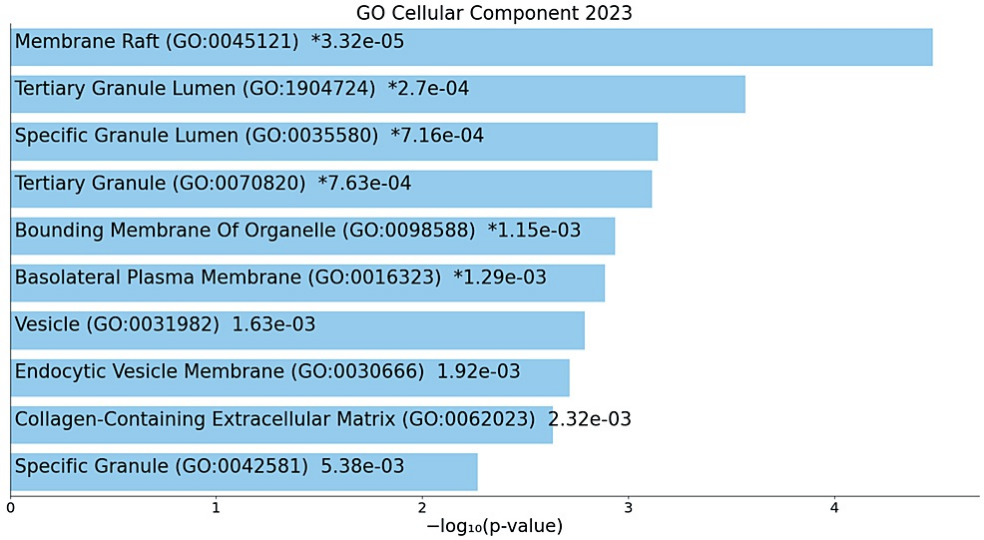
GO cellular process for downregulated genes * p < 0.05 - statistically significant GO: Gene ontology

Cellular changes downregulated terms include alterations in membrane structures, vesicular transport, and extracellular matrix composition, indicating potential disruptions in cell membrane integrity and intercellular communication (Figure 8).

**Figure 9:**
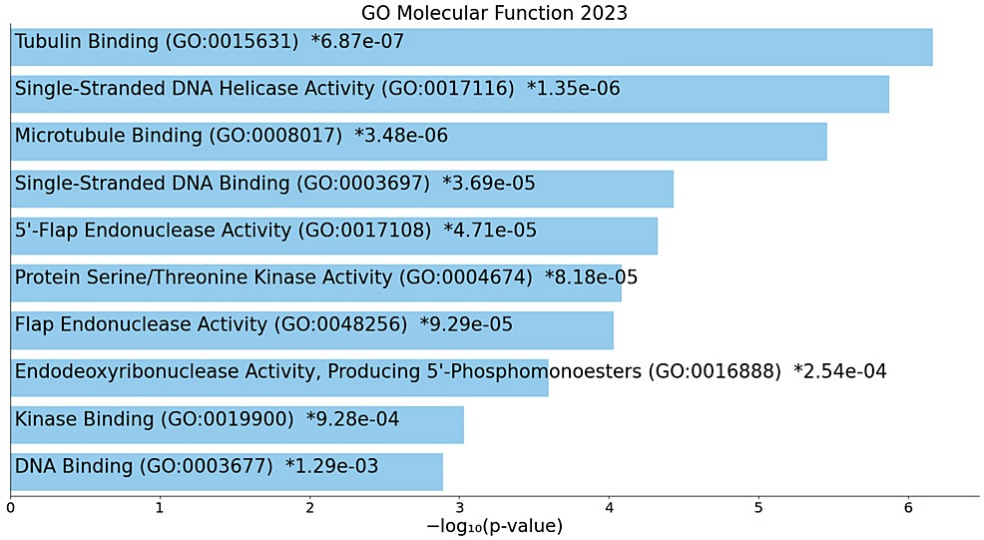
GO molecular process for upregulated genes * p < 0.05 - statistically significant GO: Gene ontology

On the molecular front, regulated terms include tubulin binding, single-stranded DNA helicase activity, microtubule binding, and protein serine/threonine kinase activity (Figure 9).

**Figure 10:**
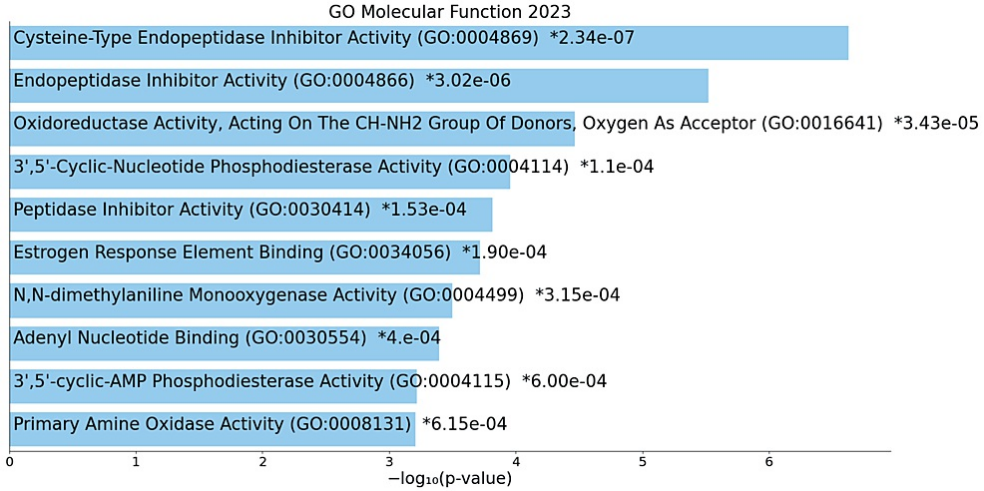
GO molecular process for downregulated genes * p < 0.05 - statistically significant GO: Gene ontology

Conversely, downregulated functions encompass cysteine-type endopeptidase inhibitor activity, oxidoreductase activity, 3',5'-cyclic-nucleotide phosphodiesterase activity, and estrogen response element binding (Figure 10).

**Figure 11:**
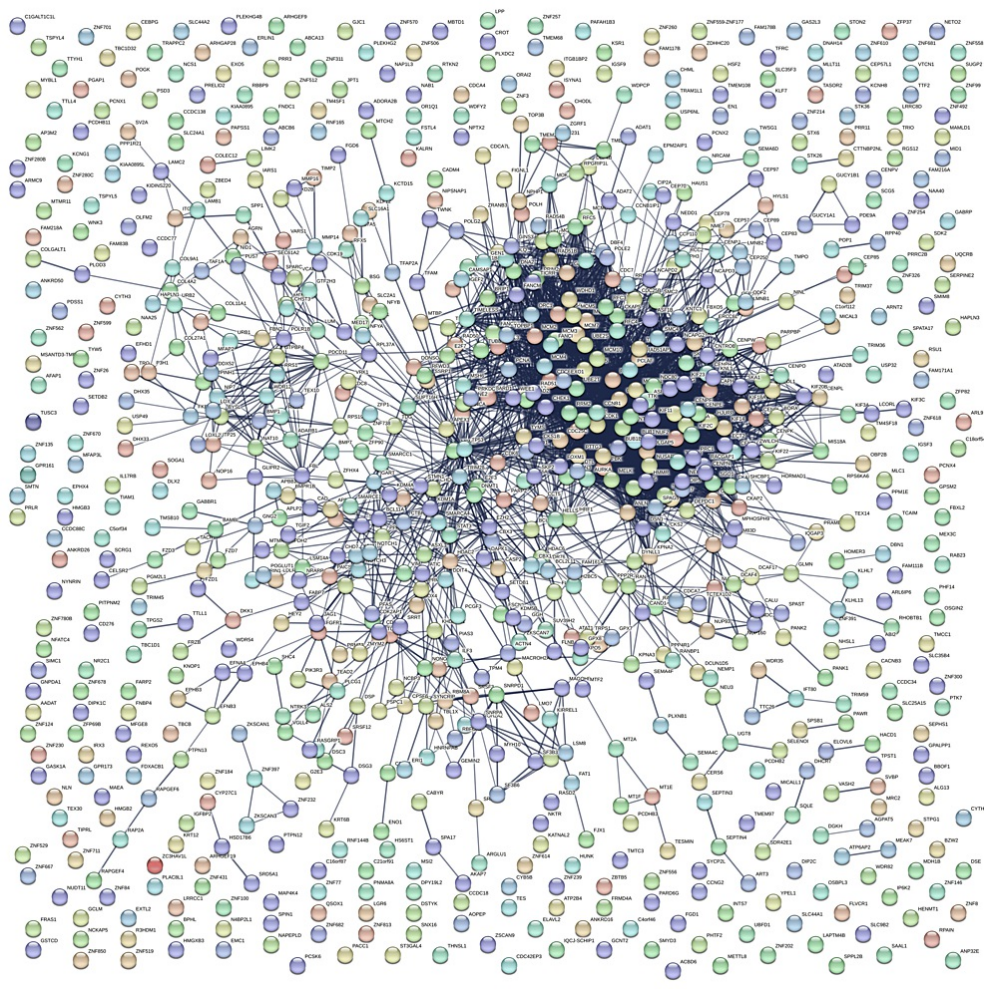
STRING PPI network In this network, nodes symbolize proteins, with each node encompassing all proteins derived from a single gene locus. Edges denote precise protein-protein associations, where colored nodes denote query proteins and their immediate interactors, and white nodes signify the second shell of interactors. The nodes are categorized into empty (unknown structure) and filled (known or predicted structure). STRING: Search Tool for the Retrieval of Interacting Genes/Proteins; PPI: Protein-protein interaction

Construction of the PPI Network:

The PPI network was constructed with 904 nodes and 4139 edges, resulting in an average node degree of 9.16. The average local clustering coefficient was calculated to be 0.391. The minimum required interaction score was set to a confidence level of 0.7, indicating the reliability threshold for the interactions within the network (Figure 11).

**Figure 12:**
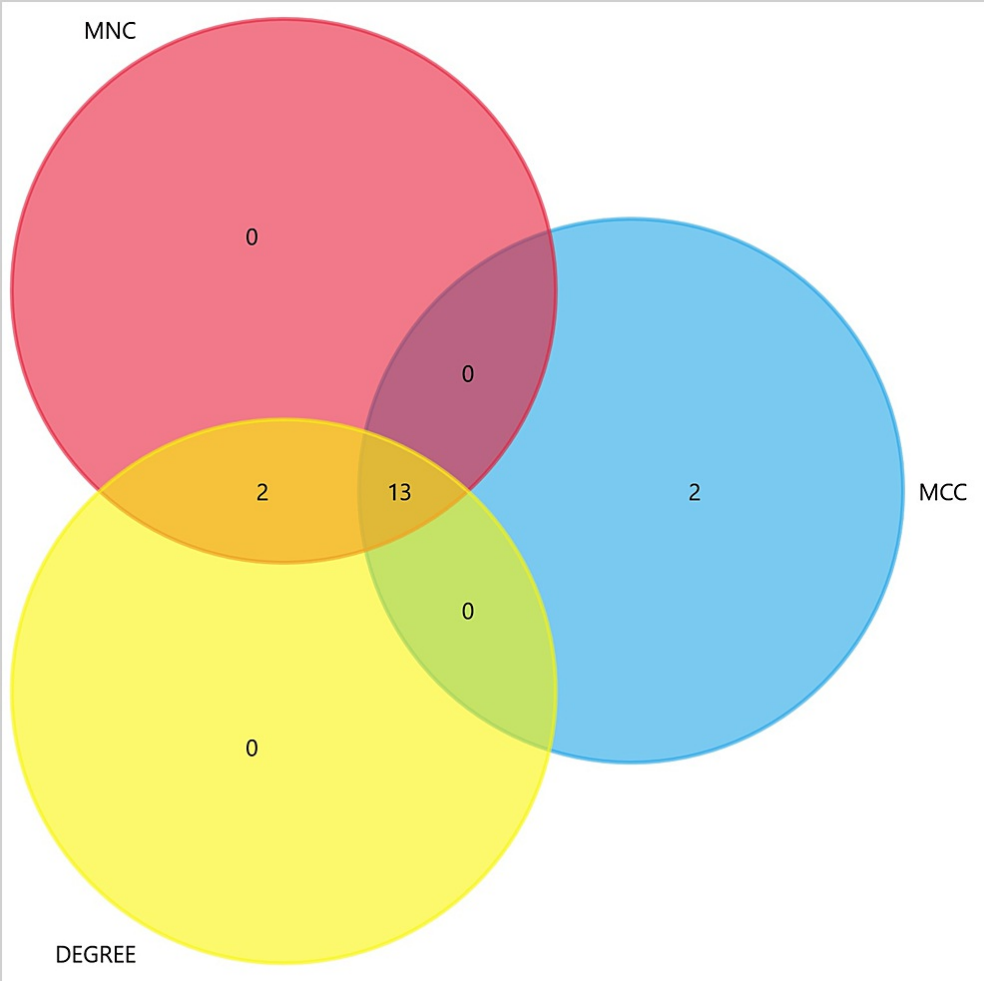
Identification of hub genes Hub genes were identified by overlapping three methods: MCC, MCN, and degree. MCC: Maximal Clique Centralit; MCN: Maximum Neighborhood Component

Selection of Hub Genes:

The identification of hub genes in the PPI network employed three distinct methods: Maximal Clique Centrality (MCC), Maximum Neighborhood Component (MCN), and degree. The CytoHubba plugin in Cytoscape, with default parameters, facilitated the selection of hub genes. The top 15 genes exhibiting the highest scores in MNC, MCC, and node degree were determined. To identify common hub genes across the three methods, an intersection was observed, and a Venn plot was generated to present the overlapping hub genes visually (Figure 12).

This analysis revealed a total of 13 hub gene including KIF11, BUB1, DLGAP5, KIF2C, BUB1B, ASPM, CDK1, CCNB1, KIF20A, TOP2A, CENPF, CCNA2, and TPX2.

**Figure 13:**
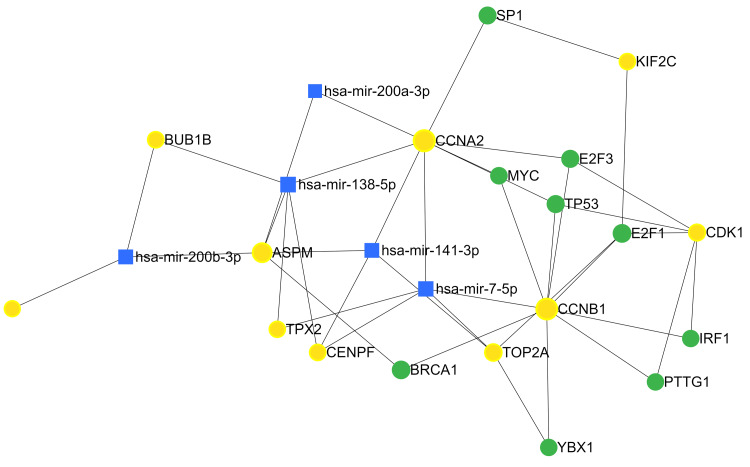
An interaction network to visualise the interaction of miRNAs and target genes Yellow nodes represent hub genes, green nodes represent transcription factors, while blue nodes were used for miRNAs. Edges represent the degree of interaction. miRNA: microRNA

miRNAs and Transcription Factors:

The comprehensive analysis of miRNAs associated with ACC has unveiled significant regulators within the intricate regulatory networks of ACC. Key miRNAs identified included hsa-mir-7-5p, hsa-mir-138-5p, hsa-mir-141-3p, hsa-mir-200b-3p, and hsa-mir-200a-3p. Simultaneously, transcription factors identified included E2F1, E2F3, TP53, BRCA1, IRF1, MYC, PTTG1, SP1, and YBX1 (Figure 13).

Hub Gene-Drug Interaction:

Among the 189 drugs identified in the drug-gene interaction analysis, 39 drugs approved by the Food and Drug Administration (FDA) were selected (Table 2).

**Table 2:** DGI with FDA regulatory approval and interaction scores The interaction scores in the table represent the strength of interaction between specific genes (e.g., TOP2A, CDK1) and their corresponding drugs, with higher scores indicating a potentially more impactful association. OCD: Obsessive-compulsive disorder; DGI: Drug-gene interaction; FDA: Food and Drug Administration

Gene	Drug	FDA Regulatory Approval	Indication	Interaction Score
TOP2A	Amsacrine	Approved		0.456393274
TOP2A	Idarubicin	Approved	Antineoplastic agents	0.04787342
TOP2A	Valrubicin	Approved	Antineoplastic agents	1.053215248
TOP2A	Podofilox	Approved	Phytogenic; keratolytic agents, antineoplastic agents	0.068687951
TOP2A	Etoposide	Approved	Antineoplastic agents	0.489652703
TOP2A	Daunorubicin	Approved	Antineoplastic agent	0.045791967
TOP2A	Dexrazoxane	Approved		0.421286099
TOP2A	Pefloxacin	Approved		0.131651906
TOP2A	Camptothecin	Approved	Antineoplastic agent	0.030091864
TOP2A	Teniposide	Approved	Antineoplastic agents	0.351071749
TOP2A	Mitoxantrone	Approved	Antineoplastic agent	0.075229661
TOP2A	Dactinomycin	Approved		0.030091864
TOP2A	Nalidixic acid	Approved		0.131651906
TOP2A	Cinoxacin	Approved		0.526607624
TOP2A	Hydroquinone	Approved		0.061953838
TOP2A	Etoposide phosphate	Approved		0.526607624
TOP2A	Digitoxin	Approved	Anti-arrhythmia agents; cardiotonic agents	0.058511958
TOP2A	Doxorubicin hydrochloride	Approved	Antineoplastic agent	0.047540966
TOP2A	Pixantrone	Approved		1.053215248
TOP2A	Enoxacin	Approved		0.263303812
TOP2A	Epirubicin	Approved	Antineoplastic agents	0.067949371
TOP2A	Daunorubicin citrate	Approved		0.526607624
CDK1	Sertraline hydrochloride	Approved	For treatment of OCD, antidepressant	0.131066786
CDK1	Clotrimazole	Approved		0.076103295
CDK1	Cinnarizine	Approved	Anti-allergic agents	0.214472923
CDK1	Rucaparib	Approved		0.047184043
CDK1	Fenofibrate	Approved	Anticholesterolaemic agent, antidyslipidaemic agent	0.09830009
CDK1	Clofibrate	Approved	Anticholesteremic agents	0.157280144
CCNA2	Genistein	Approved		0.32406623
CCNA2	Ethinyl estradiol	Approved	Contraceptive	0.99126141
CCNA2	Tamoxifen	Approved	Hormonal, antineoplastic agents	0.27625318

Notably, all of these selected drugs demonstrated an inhibitory effect.

## Discussion

The analysis of gene expression datasets GSE88804, GSE153002, and GSE36820 in ACC has unveiled a dynamic molecular landscape characterized by both upregulated and downregulated genes. Among the upregulated genes, BMPR1B, STMN1, VTCN1, GABRP, and SOX4 play vital roles in distinct cellular processes, offering insights into the unique features of ACC. BMPR1B's involvement in the bone morphogenetic protein (BMP) signaling pathway suggests a potential role in cellular growth and differentiation, aligning with the observed characteristics of cancer cells [11]. The upregulation of STMN1 implies heightened regulation of microtubule dynamics indicating increased cell cycle progression and cellular proliferation, a fundamental aspect of cancer development [12].

Conversely, downregulated genes such as TESC, ADRA1A, MYRIP, BHLHA15, and NXPE4 contribute to various cellular functions, and their decreased expression signifies potential alterations in cell adhesion, signaling, metabolism, and differentiation processes. TESC's association with cell adhesion and the extracellular matrix implies implications for the tumour microenvironment and cellular interactions, leading to altered adhesion properties in ACC cells [13]. The downregulation of ADRA1A involved in adrenergic signaling points to disruptions in cellular responses to neurotransmitters, impacting various physiological processes [14].

A closer examination of KEGG pathways unveils a comprehensive view of the molecular landscape, offering potential therapeutic targets and insights into the underlying mechanisms driving ACC development and progression. The upregulation of pathways such as the cell cycle, miRNAs in cancer, and DNA replication signifies heightened activity in cell division regulation, post-transcriptional gene regulation, and genomic stability [15]. Shared pathways with other cancers, such as breast cancer and small-cell lung cancer, suggest common molecular features, providing opportunities for cross-disciplinary insights and potential therapeutic targets. The upregulation of the p53 signaling pathway underscores the importance of tumour suppressor mechanisms in ACC, while pathways related to viral infections and hormone-mediated processes add intriguing dimensions to the molecular landscape [16]. Conversely, downregulated pathways such as salivary secretion, rheumatoid arthritis, and lipid and atherosclerosis pathways indicate systemic effects on saliva production, immune system regulation, and lipid metabolism, highlighting the need for a comprehensive understanding of ACC beyond localized changes.

In ACC, biological processes exhibit a heightened state of cellular activity, emphasizing the intricate molecular dynamics underlying the disease. The upregulation of mitotic events and DNA metabolic processes underscores the intensified cellular efforts towards accurate chromosome distribution, increased DNA-related activities, and potential immunomodulation. This suggests a dynamic and active cell division process, possibly indicating a dysregulation of cell cycle control mechanisms in ACC. Conversely, downregulated processes, including compromised responses to bacterial stimuli, suppressed inflammatory responses, and alterations in vascular smooth muscle cell proliferation, collectively imply a complex interplay between ACC cells and their microenvironment, contributing to the overall complexity of the disease [17].

A deeper exploration of cellular components sheds light on the structural and organizational changes occurring at the cellular level in ACC. Upregulated components such as spindles, microtubules, and membrane-bounded organelles paint a picture of a cellular environment characterized by heightened mitotic spindle activity, altered DNA replication processes, and potential changes in nuclear and cytoskeletal dynamics [18]. These structural alterations provide insights into the mechanisms driving ACC's cellular behavior and its interaction with the microenvironment, potentially influencing essential aspects of cell signaling and communication. Conversely, downregulated components, indicating disruptions in membrane structures, vesicles, and the extracellular matrix, further contribute to the intricate tapestry of changes, highlighting the multi-faceted nature of ACC's impact on cellular structures and interactions.

An analysis of molecular functions in ACC offers nuanced insights into specific alterations in molecular activities associated with ACC. Upregulated functions, including tubulin binding, single-stranded DNA helicase activity, kinase activity, and DNA-related activities, indicate changes in cytoskeletal dynamics, DNA maintenance, and signaling cascades, collectively contributing to the dysregulation of essential cellular processes [19]. Downregulated functions suggest alterations in enzyme activities and hormonal responses, and disruptions in nucleotide binding, influencing ACC progression. The upregulation of DNA-related activities, such as single-stranded DNA binding and endonuclease activity, highlights the significance of maintaining genomic integrity in ACC cells.

The identified hub genes, including KIF11, BUB1, DLGAP5, KIF2C, BUB1B, Abnormal Spindle Microtubule Assembly (ASPM), CDK1, CCNB1, KIF20A, TOP2A, CENPF, CCNA2, and TPX2, play pivotal roles in orchestrating various cellular processes, particularly in the regulation of cell cycle progression and mitosis. KIF11, a member of the kinesin family, contributes to mitotic spindle assembly and chromosome segregation [20]. BUB1 and BUB1B are essential components of the mitotic checkpoint, ensuring accurate chromosome segregation and preventing cell division errors [21]. DLGAP5 is involved in regulating microtubule dynamics during mitosis and cytokinesis, while KIF2C plays a role in microtubule depolymerisation [22]. ASPM is associated with mitotic spindle organization, and CDK1, along with its binding partner CCNB1, governs entry into mitosis, driving cells through the cell cycle [23]. KIF20A contributes to cytokinesis, and TOP2A is critical for DNA replication and chromosome segregation [24]. Centromere Protein F (CENPF) is involved in the formation of the mitotic spindle and chromosome alignment, while CCNA2 regulates cell cycle transitions. Finally, TPX2 plays a role in microtubule organization during mitosis. The collective functions of these hub genes underscore their significance in maintaining proper cell division and genomic stability. Dysregulation of these genes may contribute to abnormal cell proliferation, providing potential insights into the molecular mechanisms underlying ACC and suggesting avenues for targeted therapeutic interventions. Further investigation into the specific roles of these hub genes in ACC pathology is crucial for a comprehensive understanding of the disease and the development of targeted treatment strategies.

miRNAs play a crucial role in the regulatory network of gene expression in ACC, influencing various cellular processes. Among the identified miRNAs, hsa-mir-7-5p stands out for its tumour-suppressive function [25]. Known for its regulatory roles in cell proliferation, migration, and apoptosis, the potential downregulation of hsa-mir-7-5p in ACC may contribute to uncontrolled cell growth and enhanced invasiveness. This downregulation could compromise the targeting of key oncogenes, further promoting tumour development. Similarly, hsa-mir-138-5p and hsa-mir-141-3p act as tumour suppressors by inhibiting genes involved in cell proliferation, migration, and invasion. The downregulation of these miRNAs in ACC suggests a scenario where the overexpression of target genes contributes to tumour growth and metastasis. Furthermore, hsa-mir-200b-3p and hsa-mir-200a-3p play crucial roles in suppressing epithelial-mesenchymal transition (EMT) and inhibiting metastasis. The downregulation of these miRNAs in ACC may facilitate EMT, promoting tumour invasion and metastasis.

Turning to transcription factors in ACC, E2F1, and E2F3 are implicated in cell cycle regulation, with overexpression potentially driving excessive cell division and contributing to tumour growth [26]. TP53 known as the "guardian of the genome" regulates the cell cycle, DNA repair, and apoptosis. Mutations or dysregulation of TP53 in ACC may lead to uncontrolled cell growth and resistance to apoptosis [16]. BRCA1, involved in DNA repair and maintaining genomic stability, could contribute to genomic instability and promote tumourigenesis when dysregulated in ACC. IRF1, responsible for immune response regulation and tumour suppression, may impact immune surveillance mechanisms, influencing ACC progression upon altered expression.

The proto-oncogene MYC, regulating cell growth, apoptosis, and cellular transformation, is implicated in ACC [27]. Its overexpression may drive uncontrolled cell growth and inhibit apoptosis, contributing to ACC development. PTTG1, which regulates cell cycle progression and is associated with cell transformation, and SP1, a regulator of genes involved in cell growth, apoptosis, and differentiation, are also identified in ACC. Dysregulation of these factors may influence cell cycle regulation, apoptosis, and cellular behavior. Finally, YBX1, involved in transcriptional and translational regulation, contributes to cell proliferation and survival. Dysregulated YBX1 expression may impact multiple cellular processes, potentially promoting ACC progression.

The hub genes identified in ACC, including TOP2A, CDK1, and CCNA2, exhibit interactions with various drugs, shedding light on potential therapeutic interventions. TOP2A, a gene critical for DNA replication and chromosome segregation, interacts with several drugs. Amsacrine, an approved topoisomerase II inhibitor, may disrupt DNA replication and induce apoptosis in ACC cells. Idarubicin, another antineoplastic agent inhibiting topoisomerase II, could suppress ACC cell growth. Valrubicin, known for its antineoplastic properties, may interfere with topoisomerase II activity, offering a potential avenue for targeting ACC [28]. Etoposide, a topoisomerase II inhibitor, may impede DNA replication in ACC cells, contributing to its therapeutic efficacy. Additionally, dexrazoxane'sinteraction with TOP2A implies a potential role in protecting against chemotherapy-induced cardiotoxicity in ACC patients.

CDK1, a key regulator of cell cycle progression, interacts with drugs such as sertraline hydrochloride, suggesting a potential role in modulating ACC cell functions. Clotrimazole and cinnarizine, approved drugs with interactions with CDK1, indicate their potential impact on ACC cells, providing avenues for therapeutic intervention [29].

CCNA2, involved in cell cycle transitions, interacts with genistein, indicating a potential role in modulating ACC cell functions [30]. Ethinylestradiol, a contraceptive, and tamoxifen, a hormonal and antineoplastic agent, interact with CCNA2, suggesting their potential impact on ACC cells and providing avenues for therapeutic intervention.

These drug-gene interactions provide valuable insights into potential targeted therapeutic strategies for ACC, emphasizing the importance of further research to validate their efficacy and safety in clinical settings. The diverse range of drugs interacting with these hub genes highlights the complexity of ACC and the need for personalized treatment approaches based on the specific molecular characteristics of each case.

Limitations

The reliance on a restricted number of microarray datasets may limit the generalizability of findings, potentially overlooking the full heterogeneity inherent in ACC. To enhance the study's robustness, the inclusion of larger and more diverse datasets is imperative. Additionally, the predominant focus on molecular aspects creates a gap in exploring clinical correlations, impeding a holistic understanding of ACC. Integrating molecular findings with clinical data is crucial for providing context to the disease's complexity. Although bioinformatics analysis offers valuable insights, the lack of experimental validation for identified genes and pathways underscores the necessity for further confirmation.

Future directions

Research on ACC should explore promising avenues to advance our understanding and potential treatments. Multi-omics integration, involving genomics, transcriptomics, proteomics, and clinical data, can offer a comprehensive view, revealing novel biomarkers and therapeutic targets. Leveraging artificial intelligence (AI) techniques, such as machine learning algorithms, in microarray analysis can streamline data interpretation, identify subtle molecular patterns, and predict patient outcomes with higher accuracy. Functional experiments, both in vitro and in vivo, are essential for validating the functional roles identified in genes and pathways. Clinical validation with larger patient cohorts will bridge molecular insights to real-world outcomes. Delving into epigenetic modifications, specifically DNA methylation and histone modifications, can unveil additional layers of regulatory mechanisms. Immunotherapy perspectives, considering the immune microenvironment, may open new treatment avenues, emphasizing the need for a holistic and translational approach in ACC research.

## Conclusions

The analysis of gene expression patterns in ACC illuminates a dynamic molecular landscape marked by both upregulated and downregulated genes, offering insights into the intricate cellular processes governing ACC. The identified hub genes, including KIF11, BUB1, DLGAP5, KIF2C, BUB1B, ASPM, CDK1, CCNB1, KIF20A, TOP2A, CENPF, CCNA2, and TPX2, play pivotal roles in cell cycle progression and mitosis, presenting potential targets for therapeutic interventions. The dysregulation of miRNAs and transcription factors, such as hsa-mir-7-5p, E2F1, and TP53, further contributes to the molecular complexity of ACC. The analysis extends to drug-gene interactions, unveiling potential therapeutic strategies involving drugs such as amsacrine, idarubicin, and rucaparib, providing avenues for personalized treatment approaches. These findings collectively enhance the understanding of ACC's molecular mechanisms, offering a foundation for further research to advance targeted and effective therapeutic interventions for this complex cancer.
